# Research on the Rheological Properties and Diffusion Law of Coal-Based Solid Waste Geopolymer Grouting Material

**DOI:** 10.3390/ma17225433

**Published:** 2024-11-07

**Authors:** Xinyi Wang, Mei Zhou, Jinting Bai, Yue Liao, Dong Li, Boqun Zhang

**Affiliations:** 1 College of Civil Engineering, Liaoning Technical University, Fuxin 123000, China; 14741444263@163.com (X.W.); baijinting10152022@163.com (J.B.);; 2 Xinjiang Key Laboratory of Green Mining of Coal Resources, Ministry of Education, Urumqi 830023, China; 3 Engineering Research Center of Coal-Based Solid Waste Utilization in Liaoning Province, Fuxin 123000, China; 4 Department of Construction Engineering, Liaoning Provincial College of Communications, Shenyang 110122, China; 5 School of Civil Engineering, Beijing Jiaotong University, Beijing 100044, China

**Keywords:** coal-based solid waste, grouting material, rheological properties, diffusion model

## Abstract

The rheological properties and diffusion law of coal-based solid waste geopolymer grouting material (CGGM) slurry were investigated by rheological property test and diffusion theory model derivation. Based on the power-law fluid constitutive equation, a theoretical model of slurry diffusion in an inclined fissure aquifer was established, and the effect of slurry grouting time on the slurry diffusion distance under different fissure widths, fissure inclination angles, and grouting pressures were analyzed. The results show that when coal gangue:cement:fly ash = 5:4:1, sodium silicate modulus 2.0, sodium silicate content is 10%, CGGM slurry’s bleeding rate of 1%, the liquidity of 227 mm, the initial and final setting time is 412 min and 825 min, respectively, to meet the requirements of the grouting project. CGGM slurry is a typical viscosity time-varying power-law type fluid, and the slurry diffusion distance is positively correlated with the grouting pressure, fissure width, fissure inclination angle, and negatively correlated with the rheological index. The established theoretical model can provide a reference for the parameter design of CGGM slurry in grouting construction.

## 1. Introduction

China is one of the countries with the largest coal consumption in the world, with coal accounting for more than half of the primary energy production and consumption [1]. With the deepening of coal mining in China, the hydrogeological conditions in mining areas are becoming increasingly complex, and various water hazards during coal seam mining are becoming more serious. Grouting water blocking is the mainstream method for green mining in deep coal mines, and grouting materials play an important role in treating adverse geological conditions in grouting projects [2,3,4]. However, up to now, China still widely uses cement-based grouting materials, which have problems such as high production energy consumption, high mining costs, long setting time, poor slurry stability, and low cementing strength [5,6,7,8], which do not meet the requirements for high-quality development of green mines. At the same time, deep mining has the problem of long pipe distances or time in the transport of slurry, which leads to the reduction of the fluidity performance of the slurry and the surge of the risk of pipe plugging. In view of this, it is of great significance to develop a new type of high-performance and low-carbon environmentally friendly grouting material, carry out research on the rheological behavior and diffusion law of the slurry, and apply it in actual projects to help the sustainable development of green mine construction.

With the rapid development of economic construction, the amount of various types of industrial solid waste emissions is huge, and in 2021, China generated as much as 4.038 billion tonnes of bulk industrial solid waste, an increase of 0.251 billion tonnes compared with 2020, a year-on-year increase of 6.6%. Among them, the annual emission of coal gangue alone is as high as 0.829 billion tonnes, with an accumulation of about 8 billion tonnes [9,10]. The use of solid waste rich in calcium silica-alumina to prepare low-carbon cementitious materials and replace cement to prepare grouting materials has become a research hotspot due to the realization of resourceful utilization of solid waste and, at the same time, solving the problems of high permeability, high leaching concentration and high CO_2_ emission of cement-based grouting materials. In fact, there are two ways in which solid waste can be applied in the field of grouting materials. One way is to modify traditional cement-based grouting materials. For example, Xia et al. [11] proposed that cement fly ash-modified sodium silicate grouting materials by adjusting the ratio of cement, fly ash, and sodium silicate can be in to satisfy the requirements of grouting each performance. Zhang et al. [12] found that silica fume could adjust the consistency as well as the fluidity of the grouting material in the study on the optimization of admixtures based on the impact of silica fume on the water dispersion resistance of grouting materials. Liu et al. [13] studied the activity stimulation of coal gangue as a grouting material by using three methods: low-temperature calcination, grinding, and chemical activation to activate the activity of coal gangue. The best formulation of the coal gangue-lime-sulfate alkaline system was determined by an orthogonal design test method. The study showed that the working performance of cement gangue grouting material was significantly improved. Zhang et al. [14] used high-content fly ash to replace part of the cement as grouting material, analyzed its feasibility and economy, and obtained the optimal combination of on-site mining filling and grouting material by grey relational analysis. Wang et al. [15] proposed to prepare grouting filling material by using ground coal gangue powder as the main ingredient, and urea and quicklime were used as additives, and investigated the effect of grinding time and urea on coal gangue particle size and phase composition. Huang et al. [16] developed a slag-cement composite single-fluid grouting material by adding gypsum to improve the activity of slag, making the grout better thixotropy and better to play the role of seepage and leakage prevention. The other is to prepare alkaline-activated cement or geopolymer low-carbon cementitious materials as precursor raw materials under the action of alkaline exciters and to use such cementitious materials to replace traditional cement in the preparation of grouting materials, in which the solid waste raw materials in the precursor can be as high as 100%. Zhou et al. [17] successfully prepared coal-based solid waste geopolymer grouting material (CGGM) with adjustable working capacity by using mechanically activated raw coal gangue, fly ash, and other coal-based solid wastes as the main material, and sodium silicate, sodium hydroxide, and desulfurization gypsum as the excitant ligands. Zhang et al. [18] investigated the role of red mud content on the working performance of red mud-based grouting materials, and the test showed that red mud has morphological effects and hydration properties in the hydration process of the slurry, which can enhance the pumping performance of the slurry. Li et al. [19] investigated the effect of content gypsum dihydrate (NG), flue gas desulfurisation gypsum (FGD), and phosphogypsum (PG) geopolymer materials on the workability of red mud slag grouting materials with different gypsum contents. The results showed that gypsum could reduce the fluidity of the slurry and shorten the setting time. Xiang et al. [20] showed that the incorporation of limestone fines had a positive effect on the rheological properties of alkali-inspired slag/fly ash grouting materials.

In recent years, many scholars have conducted a lot of research on the mechanism of grouting slurry diffusion by combining the rheological parameters of the slurry and have achieved fruitful results. Zhang et al. [21] and others used cement-sodium silicate slurry (C-S slurry) as a typical quick-setting slurry and proposed to adopt Bingham rheological ontological model based on the yield stress and viscosity change with time to describe the flow-solid phase transition properties of quick-setting slurry, and obtained the time-varying equations of the yield stress and viscosity of the C-S slurry through indoor experiments, and established a theoretical model of the diffusion process of quick-setting slurry fissure grouting on this basis. The theoretical model of the diffusion process of slurry fissure grouting was established on this basis. Liu et al. [22] constructed a slurry diffusion model of Newtonian fluid and viscosity time-varying fluid in horizontal grouting holes of inclined fissure and verified it by comparison of model test and numerical simulation calculation and calculated and analyzed the changing law of slurry diffusion trajectory and diffusion distance in inclined fissure under different factors. Zhang et al. [23] studied the slurry diffusion process in horizontal fissure rock bodies under hydrostatic conditions, established the theoretical model of horizontal fissure slurry diffusion considering the spatiotemporal change of slurry viscosity under the condition of constant slurry injection rate, and derived the viscosity and pressure spatiotemporal distribution equation in the slurry diffusion area, and then obtained the relationship between slurry grouting pressure and grouting time and slurry diffusion radius. Wang et al. [24] took the surrounding rock of the roadway as the research object and established a fissure grouting diffusion model based on the rock composite fissure criterion by considering the stress conditions of the grouted rock and the time-varying viscosity of the slurry. Liang et al. [25] established a polymer splitting grouting diffusion model based on the rheological properties of the foam-polymer slurry and the theory of viscous hydrodynamics. Zhang et al. [26] carried out the inclined fissure dynamic water grouting test and established the inclined fissure dynamic water grouting diffusion model considering the self-weight of Bingham’s slurry through theoretical derivation. Li et al. [27] regarded the slurry as a Bingham fluid and established a slurry diffusion equation based on the slurry-rock coupling effect based on the modified cubic law and the interfacial layer eigenstructure equation. Most scholars classified the slurry flow type based on the rheological curves and the ontological equations of the fluids [28,29,30] and concluded that the conventional grouting slurries (cement slurry, clay-cement slurry, etc.) could be regarded as Newtonian or Bingham fluids when their water–cement ratios are large, but the study of diffusion models for power-law grouting fluids has rarely been reported.

On this basis, this paper explores the effects of coal gangue content, sodium silicate modulus, and sodium silicate content on the basic properties and rheological properties of the slurry for CGGM prepared by multi-component. The diffusion model of the slurry in the fissure was established to study the characteristic laws of the changes in the slurry diffusion distance and grouting pressure. This study helps to achieve the resource application of high-doped coal-based solid waste in the construction of green mines, and at the same time, solves the problems of high cost and poor fluidity performance of traditional grouting materials in the large-volume filling and grouting, which provides the basis and reference for the grouting transformation in the overtopping area of the bottom plate aquifer.

## 2. Materials and Methods

### 2.1. Raw Materials and Preparation

Relevant studies and a large number of previous experiments by the group show that the compounding of coal gangue, fly ash, slag, and ordinary silicate cement will give play to the comprehensive advantages of the close packing of the particles of multiple materials and calcium silica-aluminum coordination, which is more conducive to the development of the rheology, strength, and durability of the geopolymer [17,31,32]. The type and content of the activator ligand play a key role in the polymerization reaction. In view of this, in this paper, a new type of green, low-carbon, and low-cost grouting material, CGGM, is synergistically prepared by using coal gangue, fly ash, and ordinary silicate cement as the main raw materials, alkali silicate solution as the alkaline activator, and desulfurization gypsum as the sulfate activator.

#### 2.1.1. Raw Materials

**Figure 1:**
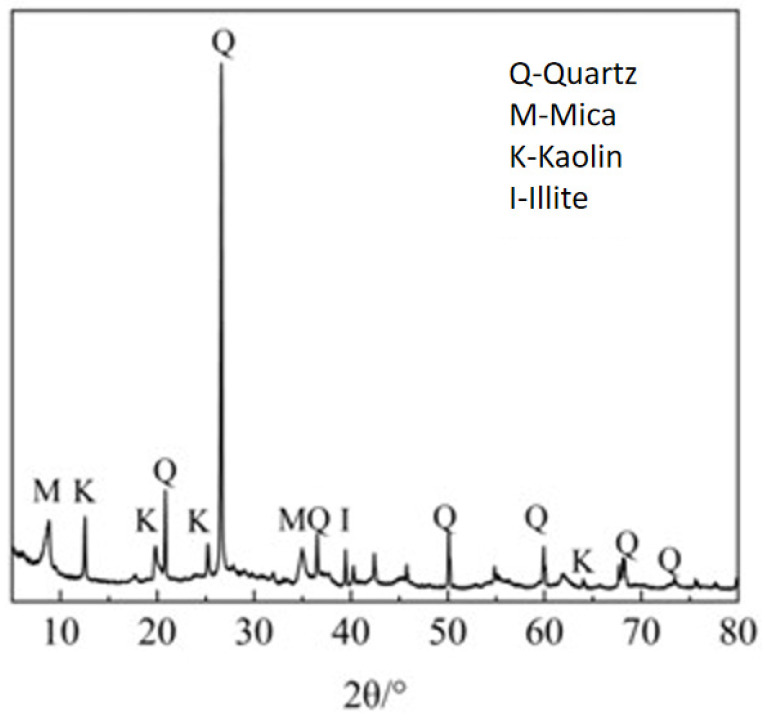
XRD pattern of coal gangue.

The coal gangue is taken from the Fengfeng mine in Handan, Hebei, and the coal gangue used is the raw coal gangue randomly sampled from the outer layer of the gangue mountain. Its chemical composition is shown in Table 1; it can be seen that the main chemical composition of the original coal gangue is SiO_2_, Al_2_O_3,_ and Fe_2_O_3_. The industrial analysis is shown in Table 2, which shows that raw coal gangue belongs to the category of low heat generation and less carbon coal gangue. The XRD pattern of coal gangue is shown in Figure 1; the main minerals in the original coal gangue are kaolin, mica, quartz, and illite. The alkaline activator will destroy the kaolin and mica in the coal gangue, the diffraction summit will be deflected to decrease, and the kaolin will gradually transform into zeolite-like.

**Figure 2:**
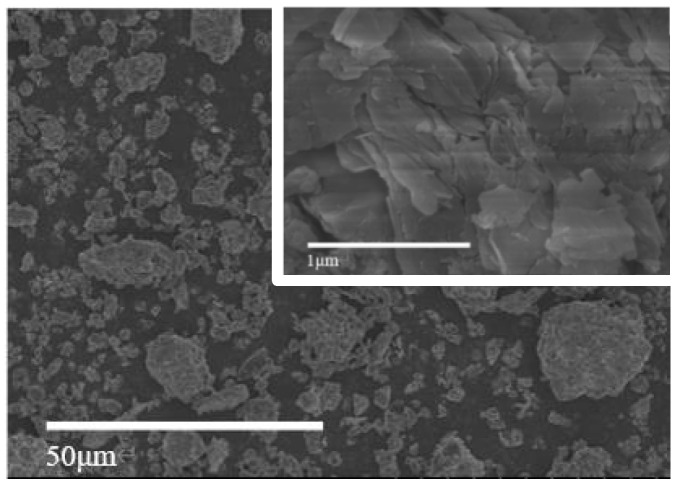
Microphotograph of coal gangue powder.

**Figure 3:**
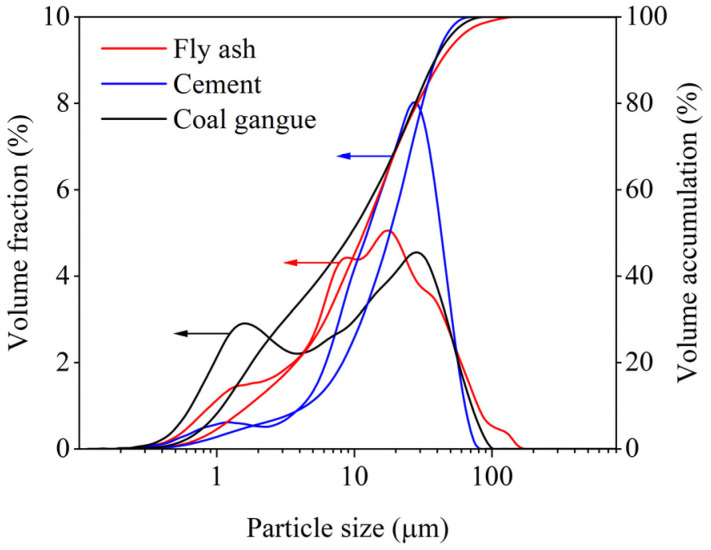
Particle size distribution of the precursor.

Firstly, the raw coal gangue is crushed into coarse aggregate by using a jaw crusher, then crushed into fine aggregate by using a sand-making machine, and finally milled by using a vertical planetary ball mill for 40 min and passed through 120 mesh square hole sieve for spare. The microphotograph of coal gangue powder is shown in Figure 2; after the coal gangue is ball-milled, there is a certain gradation between the particles, which is dominated by lamellar and irregular elliptic shapes, with some sharp angular particles with lamellar texture. The particle size distribution is shown in Figure 3.

Fly ash is class II fly ash provided by Jinfu Base Co., Ltd. (Fuxin, China). The main chemical composition is SiO_2_ and Al_2_O_3_; the apparent density is 2200 kg/m^3^, and the specific surface area is 652 m^2^/kg. Its chemical composition is shown in Table 1; the content of CaO is less than 10%, which is a low-calcium fly ash. Its chemical composition is shown in Table 1, and the distribution of the particle size is shown in Figure 3.

The cement used is 42.5-grade ordinary silicate cement of Great Eagle brand produced by Liaoning Great Eagle Cement Group Co., Ltd. (Fuxin, China), with a specific surface area of 335 m^2^/kg and a density of 3100 kg/m^3^, and its chemical composition is shown in Table 1, and the distribution of particle sizes is shown in Figure 3.

#### 2.1.2. Activator

Desulfurization gypsum, commercially available industrial grade desulfurization gypsum (2CaSO_4_·H_2_O), purity ≥ 93%, was used as the sulfate activator ligand; sodium silicate, commercially available sodium silicate, with a SiO_2_ content of 27.3%, a Na_2_O content of 8.54%, a modulus of 3.3, and a Baume degree of 38.58° Bé; and sodium hydroxide, commercially available commercially available analytically pure sodium hydroxide, with a purity of ≥96%.

#### 2.1.3. Superplasticizer

The polycarboxylic acid high-efficiency water-reducing agent provided by Jinfu Base Co., Ltd. was used at a dosage of 2%, with a water reduction rate of 20% to 25%.

### 2.2. Preparation of CGGM Slurry

**Figure 4:**
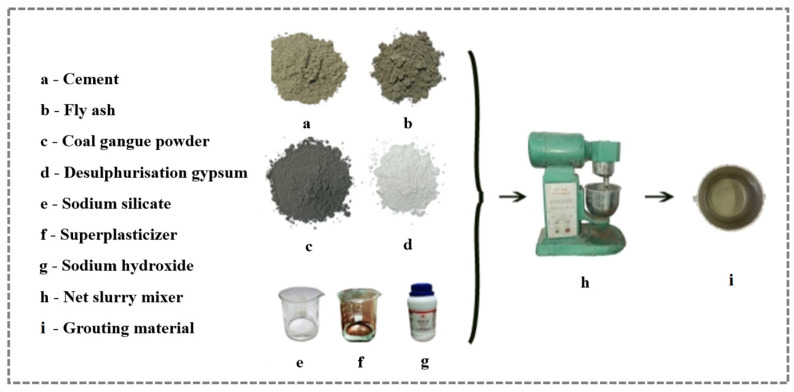
CGGM slurry preparation process.

According to the design of the test ratio, weigh the raw materials, put the powder with the activator solution and water in the net slurry mixer, and mix at low speed for 1 min; after that, add the water-reducing agent, and continue to mix at low speed for 1 min, with a stop of 30 s in the middle, and then scrape the slurry from the blades and the wall of the pot into the middle of the pot, and mix at a fast speed for 2 min. After mixing was completed, the well-mixed slurry was poured into the sample cup for rheological property testing. The CGGM slurry was prepared at room temperature, as shown in Figure 4. The test mix ratios of CGGM slurry are shown in Table 3.

### 2.3. Basic Properties Tests

Liquidity test with reference to GB/T8077-2012 [33] for test and result evaluation; setting time test with reference to GB/T1346-2011 [34] for determination; bleeding rate test with reference to GB/T25182-2010 [35] in the test method of normal pressure bleeding rate, 100 mL of slurry will be loaded into the measuring cylinder and covered with cling film (to prevent water loss), and the proportion of water precipitated from the surface of the slurry to the total volume of the slurry will be measured after 2 h.

### 2.4. Rheological Properties Test Method

The rheological properties of the grout slurry are affected by the raw material particle distribution, solid phase concentration additives, and other parameters; the rheological properties of the grout slurry in the grouting control project not only affect the diffusion range of the slurry in the geotechnical body but also has an important impact on the grouting effect. Therefore, the coal gangue powder content, sodium silicate content, and sodium silicate modulus are taken as the influencing factors to study the influence of each factor on the rheological properties of CGGM slurry.

**Figure 5:**
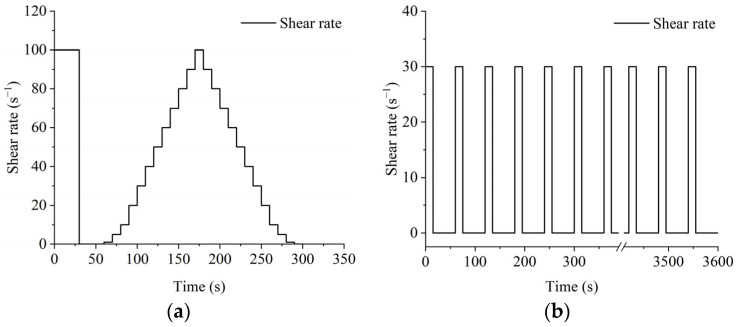
Rheological test procedure: (**a**) procedure one; (**b**) procedure two.

The American Brookfield DV3TRV rheometer (Brookfield, Brookfield, WI, USA) was used for the testing of CGGM slurry rheological properties and viscosity time-varying with the SC4 model rotor, and the control of the rheometer was completed through the Rheocaclc T software (1.2.19), and the testing procedure is shown in Figure 5.

Procedure 1: Immediately after the slurry was stirred and poured into the sample cup, it was left to stand for 30 s so that the rotor was immersed and centered in the slurry, and then it was left to stand again for 30 s before starting the measurement. A pre-shear of 30 s at a shear rate of 100 s^−1^ was used first, followed by a resting period of 30 s, after which the shear rate was first increased from 0 to 100 s^−1^ and finally reduced from 100 s^−1^ to 0 again.

Procedure 2: After predicting the viscosity range of the slurry and selecting the corresponding model of the rotor, firstly, keep the shear rate at 30 s^−1^ and shear for 15 s, and then leave it for 45 s, and then take 1 min as a cycle, and carry out a total of 60 cycles, that is, the total length of the viscosity test for the viscosity of the slurry is 60 min.

## 3. Results and Discussion

### 3.1. CGGM Slurry Basic Properties

The diffusion performance of the grouting material can be judged by the liquidity of the slurry. The construction site requires the slurry to have a good degree of liquidity but also requires the slurry to have very good stability to avoid the occurrence of water segregation. Table 3 shows the test results of the basic properties of the slurry. As can be seen from Table 3, with the increase of coal gangue content from 30% to 70%, the liquidity of CGGM slurry decreases from 260 mm to 174 mm, which is a decrease of 33.1%. Combined with Figure 3, the fineness of coal gangue powder is larger than that of cement, and the larger specific surface area decreases the free water in the system, so it reduces liquidity. When the modulus of sodium silicate increased from 1.6 to 2.4, the slurry liquidity showed a tendency to increase first and then decrease, higher ionization (low modulus) and more silicate agglomerates (high modulus) both bind free water, so leading to a decrease in liquidity. When the content of sodium silicate increased from 6% to 14%, the liquidity of the slurry increased from 217 mm to 251 mm because increasing the content of sodium silicate is equivalent to increasing the liquid–solid ratio of the slurry, which plays the role of viscosity reduction and dilution, so the liquidity of the CGGM slurry increased with the increase of sodium silicate content. The change rule of CGGM slurry setting time affected by coal gangue mixing and sodium silicate modulus is like that of slurry liquidity. The larger specific surface area of coal gangue powder makes the slurry show greater consistency under the same liquid-solid ratio, which leads to the reduction of setting time. The lower modulus of sodium silicate makes the depolymerization of Si-O Al-O bonds in the coal gangue faster and accelerates the condensation of the products, while the higher modulus improves the solubility of the precursor and provides many nucleation sites for the precipitation of the reaction products, which results in the rapid condensation and formation of the C-S-H gel accelerating the process of solidification. The slurry setting time decreases with the increase of sodium silicate content, contrary to the change rule of slurry liquidity affected by sodium silicate content, which is due to the faster reaction speed between cement and sodium silicate in CGGM, thus accelerating the reaction speed with the increase of sodium silicate, resulting in the shortening of the setting time. The CGGM slurry bleeding rate (maximum 2%) was much smaller than that of cement-based slurry (12%), indicating the stability, suspension, and anti-dispersion properties of CGGM. This can be attributed to three reasons. One is that the coal gangue contains kaolin minerals with strong water retention as well as a large specific surface area of the powder; the second is the rapid reaction between cement and sodium silicate, which generates gels such as hydrated calcium silicate to encapsulate the water in the slurry so that the slurry is too late to precipitate the water [36]; and the third is that the compound mixing of the coal gangue powder, fly ash, and cement makes the system particle gradation more reasonable.

### 3.2. CGGM Slurry Rheological Properties

#### 3.2.1. Analysis of Factors Affecting Rheological Parameters of CGGM Slurry

**Figure 6:**
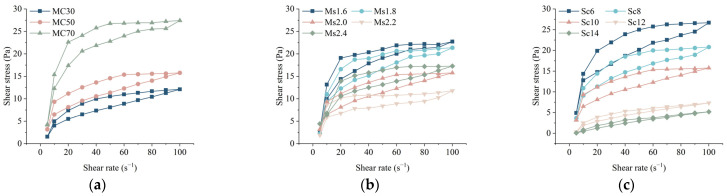
Rheology and flow pattern curve of CGGM slurry: (**a**) different coal gangue content; (**b**) different sodium silicate modulus; (**c**) different sodium silicate content.

Rheology can characterize the intrinsic relationship between the internal structure and macroscopic properties of a material [18]. Figure 6 shows the rheological profile of CGGM slurry. From Figure 6, the linear relationship between the shear stress and the shear rate of the slurry is relatively weak; the ratio of the shear stress to the shear rate decreases with the increase of the shear rate, and the slurry shows an obvious shear thinning phenomenon, and the rheological curves of the CGGM slurry are in good agreement with the power-law rheological model. The power-law rheological model is as follows:

(1) $$\tau =k\gamma^{n}$$

where *k* is the consistency coefficient, Pa·s^n^; *n* is the rheological index, no factor. The rheological index of the slurry reflects the degree of shear thinning/thickening of the slurry at each stage. When *n* is less than 1, the slurry shear thinning; when *n* is greater than 1, the slurry shear thickens. The more *n* deviates from 1, the larger the degree of shear thinning/thickening of the slurry is larger.

The tested rheological curves were fitted, and the fitted equations are listed in Table 4. From Table 4, the rheological indices of each group of slurries ranged from 0.3607 to 0.8651, indicating that the CGGM slurries are pseudoplastic shear-variable thinning fluids. A smaller rheological index means that increasing the same shear rate produces smaller shear stress, which leads to the development of shear thinning strength.

With the increase of coal gangue content, the consistency coefficient of CGGM slurry increases, while the rheological index gradually decreases, indicating that the increase of coal gangue powder content leads to an increase in the overall viscosity of the slurry and an enhancement of the degree of shear thinning. The difference is that the rheological index is coupled by the viscosity of the slurry and the stability of the reaction products, so the viscosity minimum and the rheological index minimum correspond to the difference in the sodium silicate modulus. With the increase of sodium silicate content, the consistency coefficient of the slurry decreased, and the rheological index of the slurry increased significantly, reducing the degree of shear thinning of the slurry but still showing the rheological properties of shear thinning. Among them, the Newtonian properties of the slurry Sc14 were the most significant, and the rheological properties were the closest to those of Newtonian fluids.

#### 3.2.2. Analysis of Factors Affecting Thixotropy of CGGM Slurry

**Figure 7:**
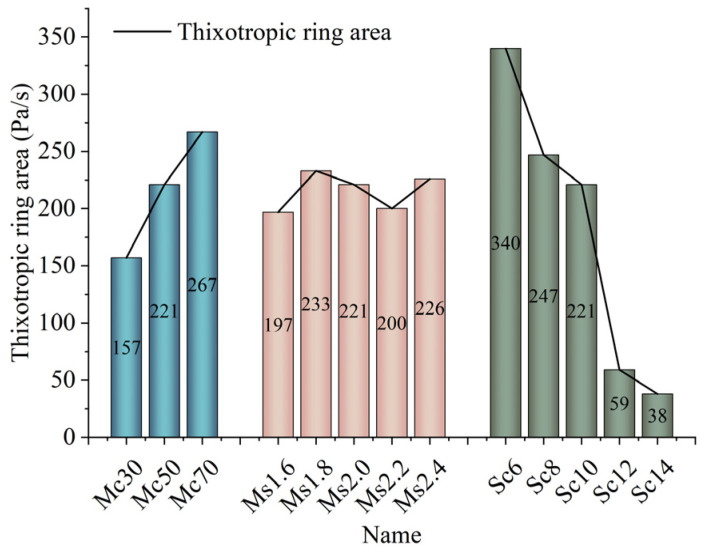
Thixotropic area of CGGM slurry.

Thixotropic ring refers to the rheological test; the sample to be tested by the shear rate gradually increased and then reduced by the upper and lower two shear stress flow curves back to the loop (see Figure 6 for details), the two curves enveloped by the area that is the thixotropic ring area. The thixotropic ring area results are shown in Figure 7. The thixotropic ring area reflects the size of the thixotropic energy storage, that is, the difference between the energy required for structure disintegration and the energy required for structure formation in the slurry. The larger the thixotropic ring area, the better the thixotropy. The slurry contains a large number of flocculated structures at a lower shear rate; the destruction of these flocculated structures requires higher shear stress, part of the flocculated structure is destroyed in the rising stage, the existence of a large number of freely suspended particles and tiny flocculated structures in the slurry reduces the required shear stress for the destruction of the tiny flocculated structures, so that in the descending section of the shear stress is less than the ascending stage. The thixotropy of the CCGM slurry is shown as follows: shear thinning, with the disappearance of external force, gradually thickens to condensation hardening. In the actual grouting process, when the slurry reaches the reinforced position, the grouting pressure gradually disappears, and the slurry becomes static and thickened, which is conducive to the retention and coagulation of the slurry, thus improving the effect of grouting and reinforcement [37]. The influence of each factor on the slurry is, in order, sodium silicate content, coal gangue content, and sodium silicate modulus.

The thixotropy of CGGM slurry gradually increases with the increase of coal gangue powder dosing. This is mainly due to the poor reactivity of coal gangue, in a relatively short period of time is not enough to generate many stabilized gel linkage particles agglomeration or flocculation structure, and the presence of coal gangue reduces the connection between cement particles. However, with the further increase of coal gangue content, the solid particles tend to be closely stacked, making up for the relative movement of particles caused by the destruction of the shear force on the agglomeration and flocculation structure, indicating that by changing the coal gangue content and then changing the slurry solid phase concentration on the thixotropy of the influence is greater than that of the coal gangue’s own reactivity on the thixotropy of the influence of the thixotropy of the coal gangue; the thixotropy of the CGGM slurry under the different modulus of the sodium silicate ring area did not show a regular. The thixotropic ring area of CGGM slurry under different sodium silicate modulus did not show a regular change; the thixotropic ring area of CGGM slurry decreased continuously with the increase of sodium silicate content. The thixotropic ring area decreased from 364 Pa/s to 38 Pa/s when the sodium silicate dosage increased from 6% to 14%, a decrease of 89.5%. This is mainly due to the fact that sodium silicate increases the colloidal particle content while increasing the average spacing of the particles in the slurry, which reduces the flocculating structure and thus decreases the thixotropic ring area.

#### 3.2.3. Analysis of the Viscosity of the Slurry and the Factors Affect

**Figure 8:**
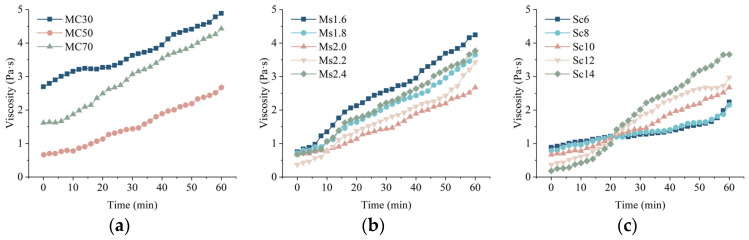
Time-varying curve of viscosity of CGGM slurry: (**a**) different coal gangue content; (**b**) different sodium silicate modulus; (**c**) different sodium silicate content.

Slurry viscosity is a key parameter of grouting engineering, which is the size of the shear stress under the action of the slurry in the unit shear rate, and it is a kind of property that hinders the flow of slurry inside the slurry, reflecting the speed of slurry flow. The evolution of the slurry over time is the result of the combined effect of chemical and physical changes in the microstructure. Figure 8 shows the time-varying curve of viscosity of CGGM under different influencing factors, which is fitted by using an exponential function like the following:

(2) $$\mu \left(t\right)=\delta e^{\beta t}$$

where *δ* is the initial viscosity of the slurry, Pa·s; *β* is the time-varying coefficient of viscosity, *t* is the grouting time, and *μ*(*t*) is the viscosity at the moment of *t*, Pa·s. The fitting equations are listed in Table 5.

As can be seen in Figure 8, there are some fluctuations in the viscosity of the CGGM slurry while it grows with time. The generated relatively stable structure in the slurry is destroyed by shear stress, which reduces the ability to resist shear stress again, while the reaction continues to take place while generating a new relatively stable structure. Therefore, the cause of the fluctuations in the viscosity time-varying curve is the coupled effect of the destruction and reconstruction of the relatively stable structures in the slurry.

As can be seen from Figure 8a, with the increase of coal gangue content, the overall time-varying viscosity of CGGM slurry shows a first decline and then a rise in the trend of change. When the dosage of coal gangue is 30%, the time-varying viscosity of CGGM slurry is the highest, which is because the reactivity of coal gangue is much smaller than cement, and at this time, due to the higher dosage of cement, its hydration rate is faster and the hydration products are more, so in the same test time, the reaction rate of the CGGM slurry and the stability of the reaction products are relatively higher; when the dosage of coal gangue is 70%, the larger specific surface area and lamellar structure of the powdered materials of coal gangue lead to a decrease and then increase in the overall trend of the time-varying viscosity. When the dosage of coal gangue is 70%, the larger specific surface area of coal gangue powder material and lamellar structure lead to the reduction of liquid film thickness on the surface of particles, increasing the contact between particles, which in turn increases the time-varying viscosity of CGGM slurry.

As can be seen from Figure 8b, the effect of sodium silicate modulus on the time-varying viscosity of CGGM slurry in the early stage (0~10 min) is not significant. This is because the sodium silicate modulus has little effect on the hydration reaction rate and reaction products in the early stage of cement, and the coal gangue and fly ash are in the stage of dissolution and depolymerization, and the polymerization reaction has not started. The effect of sodium silicate modulus on the time-varying viscosity of CGGM slurry is like the change rule of its own viscosity. With the increase of sodium silicate modulus, the time-varying viscosity of CGGM slurry shows a trend of decreasing and then increasing. The low modulus of sodium silicate solution promotes the dissolution of active substances in the particles and the reaction, and it is easy to form relatively stable products. With the increase of modulus, the interactions between particles increase, and it is easy to form cluster structures with low roundness and large diameter [38], which leads to poor dispersion and thus increases the time-varying viscosity of CGGM slurry.

As can be seen from Figure 8c, the effect of sodium silicate content on the slurry can be divided into two stages. In the first stage (0~21 min), the time-varying viscosity of CGGM slurry decreases with the increase of sodium silicate content, which is mainly due to the increase of liquid–solid ratio caused by the increase of sodium silicate content, which ultimately leads to the decrease of CGGM slurry viscosity; in the second stage (21~60 min), the time-varying viscosity of CGGM slurry increases with the increase of sodium silicate content, and the rate of viscosity growth increases faster. It originated from the presence of active SiO_2_ in the sodium silicate directly involved in the reaction and promoted the reaction, which made the reaction between [SiO_4_]^4−^ and dissolved calcium ions more active and produced more hydration products. Thus, the viscosity appeared to increase [39].

### 3.3. Theoretical Model of Grout Diffusion

#### 3.3.1. Model Assumptions

The following basic assumptions are made to derive a model for grout diffusion within the inclined fissures of the rock mass [22]:

(1) Both slurry and water are incompressible homogeneous and isotropic fluids;
(2) The ratio and flow pattern of the slurry are unchanged during the grouting process, and the flow pattern of the slurry is laminar after it enters the fissure;
(3) The static shear force on the slurry produced by the water flow is negligible;
(4) The no-slip boundary condition on the wall of the fissure is established, i.e., the slurry flow velocity on the wall is 0;
(5) The slurry diffusion mode is complete repulsive diffusion, and the dilution effect of water on the slurry at the interface of the slurry–water phase is not considered;
(6) The slurry diffuses only in the fissure, ignoring the loss of slurry caused by the infiltration of slurry into the rock body on both sides of the fissure.

#### 3.3.2. Power-Law Slurry Diffusion Equation Modeling

**Figure 9:**
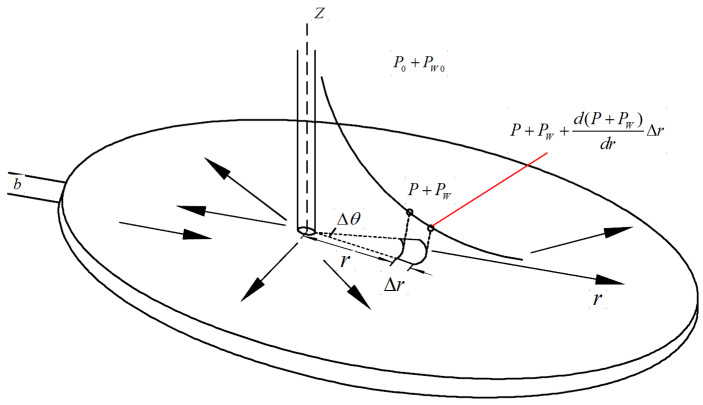
Schematic diagram of slurry diffusion in inclined fissures.

**Figure 10:**
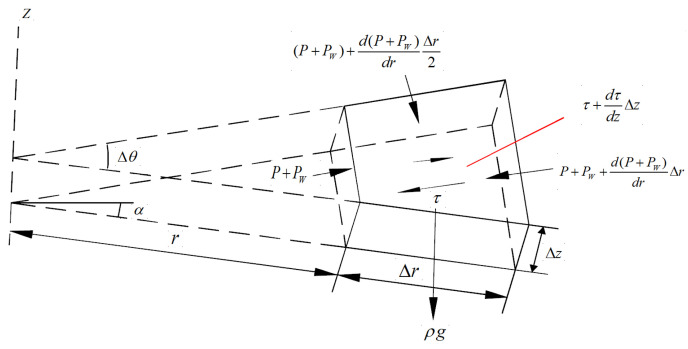
Stress analysis of slurry unit.

Based on the above assumptions, the inclined fissure can be simplified to a flat fissure that is straight, smooth, and infinitely extended. When grouting in the coal seam bottom plate, the grouting holes and fissures present the relationship of oblique intersection, vertical and parallel, among which the vertical and oblique intersection of fissures is more important for the prevention and control of the bottom plate water breakout. Therefore, the state of slurry grouting holes and fissures diagonally intersected is used for the analysis, assuming that the slurry is injected into the fissure from the grouting holes under the action of grouting pressure to flow in the plane radial parallel to the fissure surface, the grouting pressure in the grouting holes is *P*_0_, and the scouring force on the slurry caused by the flow of water is *P_w_*, and the schematic diagram of slurry diffusion in the inclined fissure is shown in Figure 9, and the force analysis of any one micrometamer is carried out from Figure 9 [40] as shown in Figure 10, and the force analysis is carried out in Figure 10 if Neglecting the static shear force generated by the water flow on the slurry and not considering the effect of the velocity change, the sum of each component force along the radial direction of the microelement body is equal to 0.

(3) $$\begin{array}{l} (P+P_{w})r\Delta \theta \Delta z-\left[(P+P_{w})+\frac{d(P+P_{w})}{dr}\Delta r\right](r+\Delta r)\Delta \theta \Delta z+\left[(P+P_{w})+\frac{d(P+P_{w})}{dr}\frac{\Delta r}{2}\right]\Delta r\Delta z\Delta \theta \\ +(\frac{d\tau }{dz}\Delta z)\frac{r\Delta \theta +(r+\Delta r)\Delta \theta }{2}\Delta r+\rho g\sin\alpha \Delta r\Delta z\frac{r\Delta \theta +(r+\Delta r)\Delta \theta }{2}=0 \end{array}$$

where *r* is the radius of slurry diffusion at any moment, unit m, ∆*r* is the increment of diffusion radius in the unit moment, ∆*θ* is the angle of microelement formation of the increment of diffusion radius in the unit moment *ρ* is the density of slurry, kg/m^3^, and *α* is the inclination angle of the fissure, °. Omitting the higher-order microelements, the collation and simplification can be obtained as in the following:

(4) $$\frac{d\tau }{dz}=\frac{d(P+P_{w})}{dr}-\rho g\sin\alpha$$

The integral with respect to *z* is obtained as in the following:

(5) $$\tau =\left(\frac{d(P+P_{w})}{dr}-\rho g\sin\alpha \right)(z+C_{1})$$

where *C*_1_ is the coefficient to be determined and substituting the boundary condition ${\left.\frac{du}{dz}\right|}_{z=0}=0\Rightarrow C_{1}=0$,

(6) $$\tau =\left(\frac{d(P+P_{w})}{dr}-\rho g\sin\alpha \right)z$$

In addition, the rheological equations for power-law fluids are typically as in the following:

(7) $$\tau =k{\left(\frac{du}{dz}\right)}^{n}$$

It is obtained by bringing Equation (7) into Equation (6) as follows:

(8) $$k{\left(\frac{du}{dz}\right)}^{n}=\left[\frac{d(P+P_{w})}{dr}-\rho g\sin\alpha \right]z$$

and integrating over *z* yields as follows:

(9) $$u=\frac{n}{n+1}{\left[\frac{1}{k}\left(\frac{d(P+P_{w})}{dr}-\rho g\sin\alpha \right)\right]}^{\frac{1}{n}}(z^{\frac{n+1}{n}}+C_{2})$$

According to the boundary condition ${\left.u\right|}_{z=\pm \frac{b}{2}}=0\Rightarrow C_{2}=-{\left(\frac{b}{2}\right)}^{\frac{n+1}{n}}$, then

(10) $$u=\frac{n}{n+1}{\left[\frac{1}{k}\left(\frac{d(P+P_{w})}{dr}-\rho g\sin\alpha \right)\right]}^{\frac{1}{n}}\left(z^{\frac{n+1}{n}}-{\left(\frac{b}{2}\right)}^{\frac{n+1}{n}}\right)$$

where *b* is the fissure width, m.

The average flow rate of the slurry is as follows:

(11) $$\bar{u}=\frac{1}{b}\int_{-\frac{b}{2}}^{\frac{b}{2}}\frac{n}{n+1}{\left[\frac{1}{k}\left(\frac{d(P+P_{w})}{dr}-\rho g\sin\alpha \right)\right]}^{\frac{1}{n}}(z^{\frac{n+1}{n}}-{\left(\frac{b}{2}\right)}^{\frac{n+1}{n}})dz=\frac{-n}{2n+1}{\left[\frac{1}{k}\left(\frac{d(P+P_{w})}{dr}-\rho g\sin\alpha \right)\right]}^{\frac{1}{n}}{\left(\frac{b}{2}\right)}^{\frac{n+1}{n}}$$

Assuming that the slurry spreads in a semicircular shape along the direction of water flow, the unit flow rate of the slurry is as follows:

(12) $$q=\pi rb\bar{u}=\pi rb\frac{-n}{2n+1}{\left(\frac{b}{2}\right)}^{\frac{n+1}{n}}{\left[\frac{1}{k}\left(\frac{d(P+P_{w})}{dr}-\rho g\sin\alpha \right)\right]}^{\frac{1}{n}}$$

where *q* is the slurry flow rate and *r* is the diffusion radius, m.

Integrating over *r* gives the following:

(13) $$P+P_{W}=\left\{\begin{array}{ll} \rho g\sin\alpha r-\frac{kq^{n}}{(1-n)\pi^{n}b^{n}{\left(\frac{n}{2n+1}\right)}^{n}{\left(\frac{b}{2}\right)}^{n+1}}r^{1-n}+C_{3}, & n\neq 1\\ \rho g\sin\alpha r-\frac{12kq}{\pi b^{3}}\ln r+C_{3}, & n=1 \end{array}\right.$$

*C*_3_ from the boundary conditions $r=r_{0}$, $P=P_{0}$, $P_{w}=P_{w0}$:

(14) $$C_{3}=\left\{\begin{array}{ll} P_{0}+P_{W0}-\rho g\sin\alpha r_{0}+\frac{kq^{n}}{(1-n)\pi^{n}b^{n}{\left(\frac{n}{2n+1}\right)}^{n}{\left(\frac{b}{2}\right)}^{n+1}}r_{0}^{1-n}, & n\neq 1\\ P_{0}+P_{W0}-\rho g\sin\alpha r_{0}+\frac{12kq}{\pi b^{3}}\ln r_{0}, & n=1 \end{array}\right.$$

where *P_w_*_0_ is the scouring force of water flow on the slurry at the grouting hole, Pa, and *r*_0_ is the radius of the grouting hole, m.

(15) $$P+P_{w}=\left\{\begin{array}{ll} P_{0}+P_{W0}+\rho g\sin\alpha (r-r_{0})-\frac{kq^{n}}{(1-n)\pi^{n}b^{n}{\left(\frac{n}{2n+1}\right)}^{n}{\left(\frac{b}{2}\right)}^{n+1}}(r^{1-n}-r_{0}^{1-n}), & n\neq 1\\ P_{0}+P_{W0}+\rho g\sin\alpha (r-r_{0})-\frac{12kq}{\pi b^{3}}\ln\frac{r}{r_{0}}, & n=1 \end{array}\right.$$

The amount of grout injected per unit of time is equal to the amount of slurry required to increase the diffusion radius during that time period as in the following:

(16) $$\int_{0}^{t}qdt=\int_{r_{0}}^{r}\pi rbdr$$

where *t* is the grouting time, s.

(17) $$qt=\frac{\pi b}{2}(r^{2}-r_{0}^{2})$$

It is obtained by bringing Equation (17) into Equation (15):

When n≠1,

(18) $$P+P_{w}=P_{0}+P_{W0}+\rho g\sin\alpha (r-r_{0})-\frac{2k{\left(r^{2}-r_{0}^{2}\right)}^{n}}{(1-n)b^{n+1}{\left(\frac{n}{2n+1}\right)}^{n}t^{n}}(r^{1-n}-r_{0}^{1-n})$$

When n=1,

(19) $$P+P_{w}=P_{0}+P_{W0}+\rho g\sin\alpha (r-r_{0})-\frac{6k(r^{2}-r_{0}^{2})}{b^{2}t}\ln\frac{r}{r_{0}}$$

Under hydrostatic conditions, the boundary conditions $r=r_{\max},\;P=0,\;P_{w}=P_{C}$ for slurry diffusion to the maximum radius are obtained by substituting the following boundary conditions:

When n≠1,

(20) $$P_{c}=P_{0}+P_{W0}+\rho g\sin\alpha (r_{\max}-r_{0})-\frac{2k{\left(r_{\max}^{2}-r_{0}^{2}\right)}^{n}}{(1-n)b^{n+1}{\left(\frac{n}{2n+1}\right)}^{n}t^{n}}(r_{\max}^{1-n}-r_{0}^{1-n})$$

where *P_c_* is the hydrostatic pressure, Pa.

When n=1,

(21) $$P_{c}=P_{0}+P_{W0}+\rho g\sin\alpha (r_{\max}-r_{0})-\frac{6k({r_{\max}}^{2}-r_{0}^{2})}{b^{2}t}\ln\frac{r_{\max}}{r_{0}}$$

#### 3.3.3. Calculation and Analysis

Taking CGGM slurry (rheological index *n* = 0.8, slurry heaviness *γ_g_* = 13.40 kN/m^3^), under the condition that the scouring force of water flow on the slurry at the grouting hole *P_w_*_0_ = 0 MPa and the radius of the grouting hole *r*_0_ = 60 mm, the influence of the other parameters as variables is analyzed. Where the time-varying properties of the slurry viscosity with the grouting time are not considered in the calculation, i.e., the viscosity is assumed to be constant (viscosity coefficient *k* = 0.2 Pa·s^n^).

**Figure 11:**
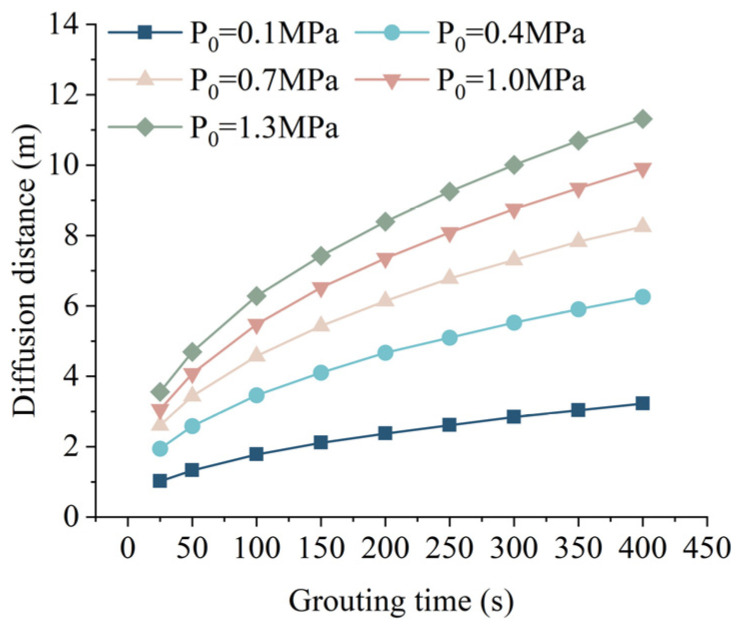
Relationship between grouting time and grouting diffusion distance under different grouting pressures.

When hydrostatic pressure *P_c_* = 0 MPa, fissure width *b* = 0.6 mm, and fissure inclination angle *α* = 30°, the relationship between the grouting time and the grouting diffusion distance of the slurry under different grouting pressures is shown in Figure 11. From Figure 11, both grouting pressure and grouting time have a positive correlation with the diffusion distance of the slurry. In other words, as the grouting pressure or grouting time increases, the diffusion distance of the slurry also increases. In practical applications, higher grouting pressure can lead to the expansion of the fissure width, which in turn facilitates the further increase in the diffusion distance of the slurry [41]. The slopes of the curves for each grouting pressure gradually decrease as grouting time increases, indicating that the time required to reach the ultimate diffusion distance also decreases with higher grouting pressure.

**Figure 12:**
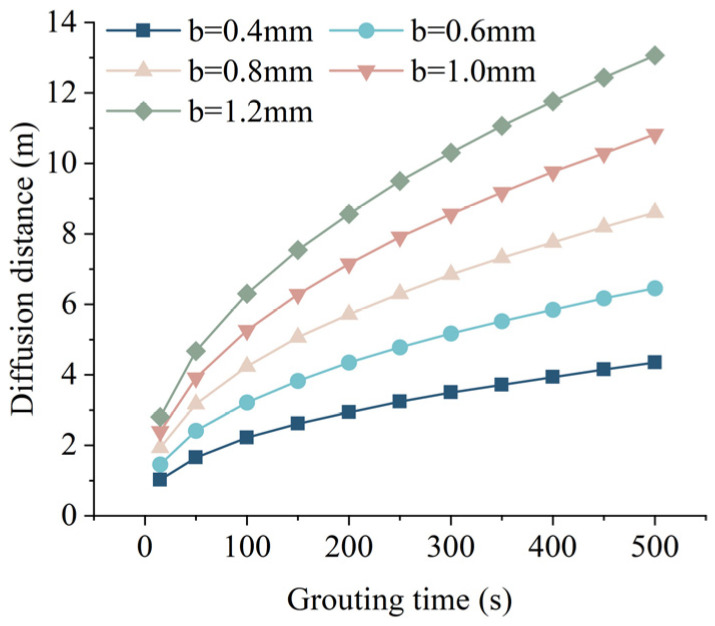
Relationship between grouting time and grouting diffusion distance under different fissure widths.

When hydrostatic pressure *P_c_* = 0.1 MPa, grouting pressure *P*_0_ = 0.5 MPa, and fissure inclination angle *α* = 30°, the relationship between grouting time and slurry diffusion distance under different fissure widths is shown in Figure 12, from Figure 12, it can be seen that, when the grouting time is the same, the larger the fissure width is, the larger the grouting diffusion distance of the slurry is. Therefore, under other conditions, the fissure width is positively correlated with the slurry diffusion distance, i.e., the larger the fissure width is, the larger the slurry diffusion distance is. However, the time required to reach the ultimate diffusion distance will be prolonged with the increase of the fissure width. Originating from the diffusion process, the slurry in the unreacted particles of the existence of deposition results in fissure blockage, reducing the diffusion capacity of the grouting slurry. The study of Zhao et al. [42] divided the horizontal hole grouting in fissure aquifer into three time periods, i.e., filling grouting, boosting penetration grouting, and high-pressure spreading grouting, in the actual grouting process, low-pressure grouting should be used in the preliminary grouting process to prevent the slurry from exceeding the design diffusion range and high-pressure splitting grouting are suitable to be used to achieve the spreading of closed fissures or micro tensioned fissures to be filled after the boosting grouting [22].

**Figure 13:**
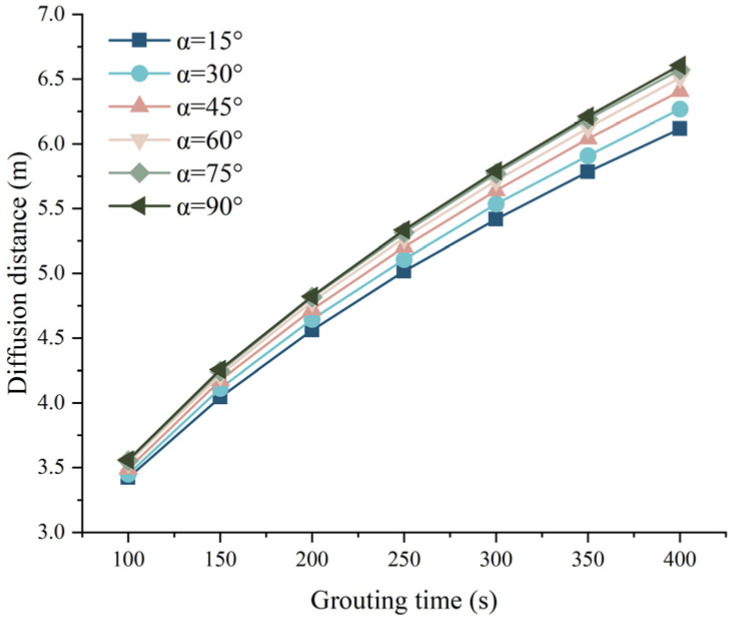
Relationship between grouting time and grouting diffusion distance under different fissure inclination angles.

When hydrostatic pressure *P_c_* = 0.1 MPa, grouting pressure *P*_0_ = 0.5 MPa, and fissure width *b* = 0.6 mm, the relationship between the grouting time and the grouting diffusion distance of the slurry under different fissure inclination angles is shown in Figure 13, as can be seen from Figure 13, under other conditions, when the grouting time is the same, the fissure inclination is positively correlated with the slurry diffusion distance, i.e., the larger the fissure inclination angle is, the larger the slurry diffusion distance is. The increase of slit inclination angle makes the influence of the self-weight effect of slurry gradually increase, which plays a positive role in the diffusion of slurry, but the ability is limited. Under the same grouting time, the influence of the fissure inclination angle on the grouting diffusion distance of the slurry is between 4.04% and 8.02%. And the increment of slurry diffusion distance at the same time tends to decrease with the increase of slit inclination angle.

#### 3.3.4. Comparison of Results of the Related Literature Models

The effect of grouting in fissure aquifers has many controlled factors, such as the angle between the fissure and the grouting hole, the grouting pressure, the grouting time, the viscosity of the slurry, etc. The grouting parameters are controllable, while the fissure parameters are objective properties of the injected medium. The results of the grout diffusion model are compared with those of the related literature to further analyze the properties of the influence of grouting parameters on the diffusion distance of CGGM slurry in inclined fissure aquifers.

Zhan et al. [40] established a diffusion model of Newtonian slurry under horizontal fissure and obtained the relationship between the diffusion distance of Newtonian slurry against the water flow direction and the grouting time under the action of a single horizontal fissure as:

(22) $$t=\frac{6\eta }{b^{2}\left(P_{0}-\frac{1}{2}\rho_{w}v^{2}-P_{c}\right)}\left(r^{2}\ln\left(\frac{r}{r_{0}}\right)-\frac{r^{2}-r_{0}^{2}}{2}\right)$$

where *η* is the slurry viscosity, Pa·s; *v* is the water flow rate, m/s.

**Figure 14:**
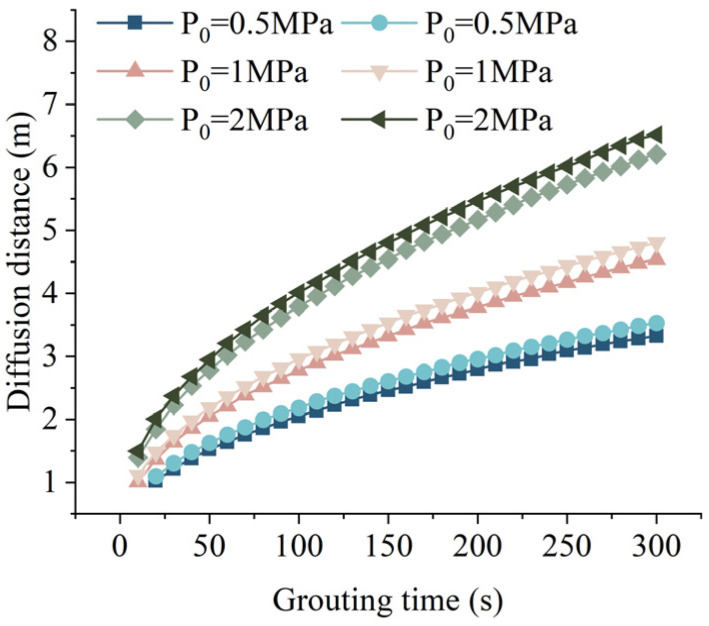
Relationship between slurry grouting time and diffusion distance [40].

Take the fissure inclination angle *α* = 0°, the radius of the grouting hole *r*_0_ = 60 mm, the fissure width *b* = 0.6 mm, the scouring force of water flow at the grouting hole on the slurry *P_w_*_0_ = 0 MPa, and the consistency coefficient *k* is equal to the slurry viscosity *η* of the Newtonian fluid. Comparing Equation (22), the diffusion model of the hydrostatic grouting (*v* = 0) with that of the Newtonian fluid in Equation (21) (with rheological index *n* = 1), the slurry grouting diffusion distance has obtained the relationship with the grouting time is shown in Figure 14. As can be seen in Figure 14, driven by different grouting pressures, slurry diffusion distance and grouting time have a parabolic relationship, and the slurry diffusion distance increases with the increase in grouting pressure; the theoretical model calculations are basically the same as the results of the model calculations in the literature [39], but due to the differences in the viscosity change rule of the slurry as well as the slurry’s own gravity, so that the difference between the slurry diffusion distance in the late stage of the slurry diffusion distance gradually obvious.

**Figure 15:**
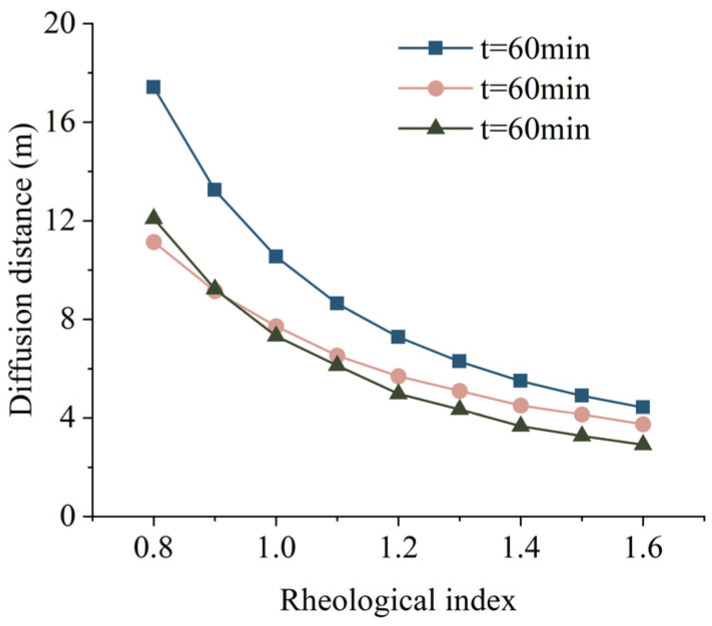
Relationship between the rheological index and slurry diffusion distance [43,44].

The relationship between the rheological index and slurry diffusion distance was studied while establishing the slurry diffusion model by Zhang [43] and Hu [44] from Central South University. Take the grouting pressure *P*_0_ = 0.5 MPa, hydrostatic pressure *P_c_* = 0.1 MPa, slurry gravity *γ_g_* = 13.40 kN/m^3^, radius of grouting hole *r*_0_ = 60 mm, fissure width *b* = 0.6 mm, fissure inclination angle *α* = 30°, scouring force of water flow at the grouting hole on the slurry *P_w_*_0_ = 0 MPa, rheological index *n* = 1, consistency coefficient *k* = 0.2 Pa·s^n^. Comparing the grouting diffusion model of the literature with Equation (20), the relationship between the rheological index and the grouting diffusion distance of the slurry when the grouting time is 60 min is obtained, as shown in Figure 15. Within the same case of grouting time, the slurry diffusion distance decreases with the increase of the rheological index, and the decrease is decreasing. The existence of yield stress may be the main reason for the obvious difference between the calculation results of the theoretical model and those in the literature.

## 4. Conclusions

In this paper, the basic properties and rheological properties of the slurry were tested, the influence of coal gangue content, sodium silicate modulus, and sodium silicate content on the basic properties and rheological properties of CGGM slurry were analyzed, and the theoretical model of the diffusion of the power-law fluid slurry in inclined fissure aquifers was established to study the characteristic laws of the changes in the slurry diffusion distance and the grouting pressure, etc., so as to provide a basis for the bottom version of the over-above-area grouting renovation of the aquifer and to provide reference for the bottom version of the aquifer. The specific conclusions are as follows:

(1) The CGGM slurry has good stability, suspension, and anti-dispersion, and its liquidity, setting time, and bleeding rate meet the requirements of grouting construction. The CGGM slurry is a shear-thinning power-law fluid; the increase of coal gangue powder content will increase the shear thinning degree of the slurry, and the increase of sodium silicate content will reduce the shear thinning degree of the slurry, of which the sodium silicate content is 14%, CGGM slurry is close to the Newtonian fluid. The slurry’s thixotropy rises with increasing coal gangue powder content and falls with increasing sodium silicate content, with the sodium silicate modulus having no discernible influence;
(2) The CGGM slurry is a typical viscosity time-varying fluid and a unified exponential function ($\mu (t)=\delta e^{\beta t}$) can be used to express the viscosity time-varying curve of this fluid. The time-varying viscosity of the slurry with the increase of coal gangue powder content and sodium silicate modulus shows a tendency to decrease and then increase. The effect of sodium silicate content on the time-varying viscosity is divided into two stages according to the time; the first stage decreases with the increase of sodium silicate content, and the later stage is the opposite;
(3) The theoretical model of slurry diffusion of grouting slurry in a single inclined fissure aquifer is established after theoretical derivation based on the intrinsic equation of power-law fluid. Theoretical and numerical analyses demonstrate that the distance of slurry diffusion is negatively correlated with the rheological index and positively correlated with the grouting pressure, fissure width, and fissure inclination angle;
(4) The established theoretical model of slurry diffusion can be solved, and the reasonableness of the model can be verified with the data of references. It is shown that the application of the theoretical model derived in this paper can better describe the slurry diffusion law of grouting along any inclined fissure aquifer, further illustrating the reasonableness and reliability of the theoretical model and providing a theoretical basis for the subsequent grouting project.

## Figures and Tables

**Table 1:** Main chemical components of the precursor (w%).

Composition	SiO_2_	Al_2_O_3_	Fe_2_O_3_	MgO	CaO	K_2_O	Na_2_O	SO_3_
Coal gangue	57.74	30.58	4.31	1.00	0.20	2.76	1.10	0.16
Fly ash	62.02	11.29	5.19	1.56	6.07	1.63	0.13	1.04
Cement	22.72	7.74	4.72	2.71	56.84	-	-	2.72

**Table 2:** Industrial analysis results of coal gangue.

Test Items	Results	Test Items	Results	Test Items	Results
Air-dried Moisture /*M*_ad_(%)	0.9	Total Moisture /*M*_t_(%)	1.1	Dry basis full sulfur /*S*_t,d_(%)	0.35
Dry Basis Ash /*A*_d_(%)	89.3	Dry Basis Gross Calorific Value /*Q*_gr,d_(MJ/Kg)	0.72	Air-dried fixed carbon /*FC*_ad_(%)	3.39
Dry Ash-free Volatile /*V*_daf_(%)	68.02	Received base low Calorific Value /*Q*_neg,ar_(MJ/Kg)	0.51	Cinder Bondability /*CB*	2
Deflection Temperature/*DT*(°C)	1460	Softening Temperature/*ST*(°C)	>1500		

**Table 3:** CGGM slurry mixing ratios and their basic properties.

Name	Coal Gangue Content/%	Cement Content/%	Fly Ash Content/%	Sodium Silicate Modulus	Sodium Silicate Content/%	Liquidity /mm	Setting Time/min		Bleeding Rate/%
							Initial	Final	
Mc30	30	60	10	2.0	10	260	633	1384	2%
Mc50	50	40	10	2.0	10	227	412	825	1%
Mc70	70	20	10	2.0	10	174	182	583	0%
Ms1.6	50	40	10	1.6	10	192	445	1058	0%
Ms1.8	50	40	10	1.8	10	197	473	1096	1%
Ms2.0	50	40	10	2.0	10	227	412	825	1%
Ms2.2	50	40	10	2.2	10	229	469	1090	1%
Ms2.4	50	40	10	2.4	10	214	451	1039	0%
Sc6	50	40	10	2.0	6	217	680	1254	1%
Sc8	50	40	10	2.0	8	223	616	1174	1%
Sc10	50	40	10	2.0	10	227	412	825	1%
Sc12	50	40	10	2.0	12	238	326	877	0%
Sc14	50	40	10	2.0	14	251	255	659	0%

Note: The percentages in the table are the mass ratios of the precursors; the fixed Na_2_O content and desulfurization gypsum dosing are 3% and 5% of the total powder mass, and the water–cement ratio is 0.8.

**Table 4:** Rheological equation and parameters of CGGM slurry.

	Index	Rheological Equation	*k*/Pa·s^n^	*n*	R^2^
Name					
Mc30		$\tau =1.0794\gamma^{0.5221}$	1.0794	0.5221	0.9862
Mc50		$\tau =2.1713\gamma^{0.4288}$	2.1713	0.4288	0.9847
Mc70		$\tau =5.1683\gamma^{0.3705}$	5.1683	0.3705	0.9106
Ms1.6		$\tau =3.8956\gamma^{0.3926}$	3.8956	0.3926	0.9095
Ms1.8		$\tau =3.1064\gamma^{0.4244}$	3.1064	0.4244	0.9343
Ms2.0		$\tau =2.1713\gamma^{0.4288}$	2.1713	0.4288	0.9847
Ms2.2		$\tau =2.0807\gamma^{0.3607}$	2.0807	0.3607	0.9049
Ms2.4		$\tau =3.0024\gamma^{0.3779}$	3.0024	0.3779	0.9704
Sc6		$\tau =4.3445\gamma^{0.3909}$	4.3445	0.3909	0.9567
Sc8		$\tau =3.2273\gamma^{0.4021}$	3.2273	0.4021	0.9637
Sc10		$\tau =2.1713\gamma^{0.4288}$	2.1713	0.4288	0.9847
Sc12		$\tau =0.4182\gamma^{0.6241}$	0.4182	0.6241	0.9837
Sc14		$\tau =0.0988\gamma^{0.8651}$	0.0988	0.8651	0.9949

**Table 5:** Time-varying viscosity equation of CGGM slurry.

Name	Time-Varying Viscosity Equation	R^2^	Name	Time-Varying Viscosity Equation	R^2^
Mc30	$\mu (t)=2.7611e^{0.00016t}$	0.9856	Ms2.4	$\mu (t)=1.0167e^{0.00038t}$	0.9559
Mc50	$\mu (t)=0.7338e^{0.00037t}$	0.9853	Sc6	$\mu (t)=0.8997e^{0.00020t}$	0.9009
Mc70	$\mu (t)=1.6171e^{0.00030t}$	0.9414	Sc8	$\mu (t)=0.8681e^{0.00022t}$	0.9655
Ms1.6	$\mu (t)=1.2517e^{0.00035t}$	0.9490	Sc10	$\mu (t)=0.7338e^{0.00037t}$	0.9853
Ms1.8	$\mu (t)=0.9856e^{0.00037t}$	0.9721	Sc12	$\mu (t)=0.7123e^{0.00043t}$	0.9029
Ms2.0	$\mu (t)=0.7338e^{0.00037t}$	0.9853	Sc14	$\mu (t)=0.6372e^{0.00052t}$	0.9133
Ms2.2	$\mu (t)=0.7502e^{0.00043t}$	0.9242			

## Data Availability

The original contributions presented in the study are included in the article, further inquiries can be directed to the corresponding author.
